# Editorial: Emerging views and players in neuronal calcium signaling: synaptic plasticity, learning/memory, aging and neuroinflammation

**DOI:** 10.3389/fncel.2023.1197417

**Published:** 2023-04-17

**Authors:** Tatiana Adasme, Cecilia Hidalgo, Rodrigo Herrera-Molina

**Affiliations:** ^1^Faculty of Medicine, Biomedical Research Institute (BNI), Universidad de Chile, Santiago, Chile; ^2^Clinical Hospital and Laboratory of Translational Psychiatry, Department of Neuroscience and Department de Psychiatry North, Center for Advanced Clinical Investigation (CICA), Universidad de Chile, Santiago, Chile; ^3^Department of Neuroscience, Physiology and Biophysics Program, Institute of Biomedical Sciences (ICBM), Faculty of Medicine, Center for Exercise, Metabolism and Cancer (CEMC), Universidad de Chile, Santiago, Chile; ^4^Laboratory of Synaptic Signaling, Leibniz Institute for Neurobiology, Magdeburg, Germany; ^5^Centro Integrativo de Biología y Química Aplicada, Universidad Bernardo O‘Higgins, Santiago, Chile

**Keywords:** RyR2 Ca^2+^release channel, LTP, Ca^2+^-induced enzyme activities, plasma membrane Ca^2+^-ATPases, retrograde amnesia

This Research Topic presents cellular and molecular mechanisms that shape cytoplasmic Ca^2+^ signals and Ca^2+^-promoted signaling pathways. In particular, the original articles harboured by this Topic describe how mechanisms regulating Ca^2+^ responses underlie the remarkable capacity of neurons and astrocytes to undergo changes in response to physiological activation and plasticity, promote learning and memory, or mediate memory loss, cell death, or oxidation-associated neurodegeneration.

The increment in cytoplasmic Ca^2+^ concentration is a powerful signal for the initiation of a variety of cellular processes in neurons and glial cells. As an example, astrocytes can sense their environment and respond quickly to the changes caused from nearby dying cells, as described in this Topic by Gomez-Godinez et al.. These authors used laser nano-surgery to trigger cell photolysis while evaluating the response of neighboring astrocytes to this treatment. Interestingly, responsive astrocytes display cytoplasmic Ca^2+^ transients largely dependent on the endoplasmic reticulum and mediated by inositol 1,4,5-trisphosphate (IP_3_) and Ryanodine receptors (RyR) Ca^2+^ channels, which are distinguishable from the spontaneous Ca^2+^ oscillations occurring in resting neuronal cells.

In neurons, Ca^2+^-dependent signaling pathways can be switched on by Ca^2+^ influx triggered by depolarizing stimuli or neurotransmitter release. The resulting cytoplasmic Ca^2+^ signal is rapidly coordinated with and amplified by Ca^2+^ release from intracellular stores through Ca^2+^-induced Ca^2+^ release (CICR) by type-2 Ryanodine Receptor (RyR2) Ca^2+^ release channels. In this Topic, Valdés-Undurraga et al. report that LTP induction promotes RyR2 Ca^2+^ release channel expression and that suppression of RyR Ca^2+^ release channel activity abolishes LTP induction and the enhanced expression of these Ca^2+^ release channels in CA3-CA1 synapses in hippocampal slices. Notably, training in the Morris water maze induces the expression of RyR2 Ca^2+^ release channels in rats. Based on these findings, the authors propose that the increments in the protein content of RyR2 Ca^2+^ release channels play a significant role in hippocampal synaptic plasticity and spatial memory consolidation.

Regarding Ca^2+^-induced enzyme activities, Vergara et al. report that Ca^2+^-dependent Calmodulin Kinase II (CaMKII) plays a key role in homeostatic synaptic plasticity processes, which are triggered by prolonged changes in neural activity and allow neuronal networks to operate within functional ranges. These authors report that transient preincubation with a specific CaMKII inhibitor either blocks or occludes amplitude and frequency of miniature excitatory postsynaptic currents (mEPSCs) in synapses of CA1 pyramidal neurons in hippocampal slices, an indication of the involvement of CaMKII in homeostatic synaptic plasticity processes. Vergara et al. propose that their results may contribute to understand fast-developing homeostatic or pathological functional changes after brain injury.

Along the same lines, Zhuang et al. report that inhibition of CaMKII and N-methyl-D-aspartate (NMDA) receptors in the lateral habenula, impact allodynia and anxiety-like behavior in mice. Moreover, activation of NMDA receptors in the lateral habenula increases the expression of the phosphorylated forms of the NR2B subtype of NMDA receptors and CaMKII and induces orofacial allodynia and anxiety-like behaviors in naive mice. Based on these findings, Zhuang et al. suggest that suppressing CaMKII activity in bilateral habenula neurons represent a novel strategy for pain treatment and anxiety associated with trigeminal neuralgia.

Ca^2+^ pumps located at the plasma membrane act rapidly and efficiently to remove Ca^2+^ from the cytoplasm and to switch off Ca^2+^ signals. The plasma membrane Ca^2+^-ATPases (PMCAs) are P-type integral membrane pumps with ATPase-consuming activity that expulses cytoplasmic Ca^2+^ toward the extracellular space, in isoelectric exchange for extracellular H^+^ (Brini et al., 2013). Still, it remains unknown what is the relevance of the large PMCA expression repertoire by four genes encoding four PMCA forms, PMCA1-4, each of them with dozens of alternatively spliced variants. In this issue, Corradi et al. compare the structure and activity of two splicing variants of PMCA4, the ubiquitously expressed PMCA4xb and PMCA4zb, which expression is restricted to heart muscle and brain. They report that PMCA4z is more active than PMCA4xb, since it displays higher apparent Ca^2+^ affinity, and is highly sensitive to acidic lipids. This study provides evidence for PMCA specialization in excitable cells (Strehler, 2013).

The expression and protein stability of PMCA1-4 is critically dependent of the interaction with neuroplastin, a cell recognition molecule expressed by neurons in the brain (Bhattacharya et al., 2017; Herrera-Molina et al., 2017; Schmidt et al., 2017). In this Topic, Montag reviews this interaction and connects it with retrograde amnesia of associative memories, one of the unique phenotypes displayed after neuron-specific neuroplastin ablation in adult mice. He proposes that “loss of neuroplastin allows PMCA degradation resulting in inappropriately high Ca^2+^ levels which interfere with signal transmission....changing network activities that may finally impair retrieval or result in loss of the memory trace.” Further connections between neuroplastin-PMCA loss with important functions in behavior and synapse transmission and plasticity should be explored, especially considering the functional interplay of neuroplastin-PMCA with ionotropic glutamate receptors of the NMDA type (iGluNRs) (Stawarski et al., 2020; Malci et al., 2022) and with AMPA type (iGluARs) (Jiang et al., 2021; Malci et al., 2022).

The article by Junghans et al. deals with Amyotrophic lateral sclerosis (ALS), an incurable neurodegenerative disease characterized by loss of motor neurons in the cerebral cortex, brainstem, and spinal cord. These authors argue that while oxidative stress signs in postmortem neuronal tissue, cerebrospinal fluid, plasma, and urine of ALS patients are evident, information is lacking on specific processes in motor neurons. Hence, they investigated the relevance of reactive oxygen species (ROS) detoxification mechanisms in the Wobbler mouse ALS model. They report elevated ROS and DNA damage response proteins (p53bp1 and gH2ax) levels in cultured Wobbler spinal cord motor neurons. They also report altered expression of antioxidant molecules in the spinal cord of Wobbler mice and propose that maintenance of redox homeostasis may play a key role in the therapy of ALS. Albeit Ca^2+^ signaling was not directly tested in this article, the significant crosstalk between Ca^2+^ and ROS signaling (Hidalgo and Donoso, 2008) makes very likely the occurrence of altered Ca^2+^ signaling in motor neurons of the ALS rodent model.

In conclusion, the original articles presented in this Topic are clear examples of how cytoplasmic Ca^2+^ signals and Ca^2+^-promoted signaling pathways control the response of neurons and astrocytes under physiological or pathological conditions. Alterations in these key regulatory mechanisms could result in oxidative damage, abnormal synapse function, and ultimately, memory loss or neurodegeneration.

## Author contributions

TA, CH, and RH-M were guest editors of the Research Topic, emerging views and players in neuronal calcium signaling, synaptic plasticity, learning/memory, aging and neuroinflammation, and wrote this editorial. All authors contributed to the article and approved the submitted version.
